# Assessment of the relationship between generalized convulsive epilepsy and systemic inflammatory regulators: a bidirectional Mendelian randomization study

**DOI:** 10.3389/fneur.2023.1206290

**Published:** 2023-07-03

**Authors:** Shengnan Wang, Tengfei Su, Shuyan Pang, Jianglong Wang, Yue Lang, Mingqin Zhu, Li Cui

**Affiliations:** ^1^Department of Neurology, The First Hospital of Jilin University, Changchun, China; ^2^First Operating Room, The First Hospital of Jilin University, Changchun, China

**Keywords:** generalized convulsive epilepsy, epilepsy, inflammation, Mendelian randomization, instrumental variables

## Abstract

**Background:**

Generalized convulsive epilepsy (GCE), an important subtype of epilepsy, is a syndrome of neuronal dysfunction characterized by diffuse abnormal discharge of neurons within the brain. Compounding evidence suggests a correlation between epilepsy and inflammatory factors, for instance, cyclooxygenase-2, interleukin-1β, and interleukin-6. Elevated levels of inflammatory factors have been observed in patients with epilepsy and several animal models. Therefore, inflammation may be closely associated with the pathogenesis and progression of GCE. However, the cause-and-effect relationship between the two is difficult to determine because of small sample sizes and confounding factors.

**Methods:**

To test for causality of the 41 cytokines on GCE, we conducted a two-sample Mendelian randomization (MR) based on the largest and latest genome-wide association study (GWAS) involving 290 cases and 453,521 European controls and a GWAS meta-analysis consisting of 41 cytokines from 8,293 individuals.

**Results:**

R confirmed a bidirectional causal link between cytokines and GCE. Genetically predicted increased levels of hepatocyte growth factor and decreased levels of eotaxin and interleukin-18 are associated with an increased risk of GCE (OR = 1.904, 95% CI = 1.019–3.561, *p* = 0.044; OR = 0.641, 95% CI = 0.417–0.984, *p* = 0.042; OR = 0.482, 95% CI = 0.251–0.927, *p* = 0.046). Furthermore, the presence of GCE is related to an increase in levels of multiple cytokines, such as macrophage inflammatory protein-1α, interleukin-12p70, interleukin-17, interleukin-1 receptor antagonist, and basic fibroblast growth factor (OR = 1.038, 95% CI = 1.005–1.073, *p* = 0.024; OR = 1.031, 95% CI = 1.009–1.054, *p* = 0.006; OR = 1.027, 95% CI = 1.002–1.053, *p* = 0.037; OR = 1.037, 95% CI = 1.003–1.072, *p* = 0.032; OR = 1.032, 95% CI = 1.000–1.066, *p* = 0.048; OR = 1.025, 95% CI = 1.003–1.048, *p* = 0026).

**Conclusion:**

A bidirectional causal link existed between inflammation and GCE. Detecting significantly altered factor concentrations may be of great significance for screening GCE and predicting their occurrence. Moreover, available pharmacological treatments for GCE are focused primarily on suppressing seizures. In future, altering the concentration of these cytokines in the body through targeted anti-inflammatory therapy to modify the epileptogenic mechanism and prevent the recurrence and refractoriness of GCE may become the key to new treatments.

## 1. Introduction

Approximately 50 million people have epilepsy worldwide, with two million people suffering annually. In a previous meta-analysis of epilepsy, Sanjeeb et al. (1) reported that generalized convulsive epilepsy (GCE) patients accounted for 23.8% of those with epilepsy of all ages. Given the resistance characteristics of GCE, early screening and prevention are necessary for GCE's identification and management. Understanding the etiology and pathogenesis of GCE is essential for its prevention and treatment. However, the exact pathophysiological mechanisms underlying GCE remain unknown. Previous studies have suggested that this may be related to an imbalance between neuronal excitation and inhibition, which leads to the burst firing of increased synchronous electrical neuronal activity. Moreover, this phenomenon is occasionally enhanced in response to physiological stimuli. Furthermore, because of the increased hypersynchronous activity of normal neurons, normal cortical areas have stronger activation and synchronization, allowing this process to spread more vigorously to other cortical areas. With the progressive increase in cortical synchrony, the activation of subcortical or brainstem structures by descending projections may be key to transforming non-convulsive disorders into persistent GCE (2).

Several studies have reported the association between inflammation and epilepsy (3). There is evidence that seizures are related to pro-inflammatory cytokines, particularly interleukin-1β (IL-1β), IL-6, and tumor necrosis factor-α (TNF-α) (4). As an important subtype of epilepsy, we suggested that the occurrence of GCE may be significantly correlated with inflammation. Moreover, reverse causation and residual confounding factors are common biases in conventional observational studies, making it challenging to clarify a causal relationship between inflammatory factors and GCE. Nevertheless, causal evidence can be determined using Mendelian randomization (MR).

Mendelian randomization (MR) utilizes genetic variants as instrumental variables (IVs) to explore the causal effects of exposure on outcomes of interest, which can overcome the limitations of observational research (5). As genotypes are randomly assigned during gamete fusion, MR analysis is less likely to be affected by reverse causality and confounding factors, similar to a randomized controlled trial. Hence, we performed a two-sample bidirectional MR study to assess the relationship between inflammatory factors and the risk of GCE.

## 2. Material and method

### 2.1. Study design

**Figure 1:**
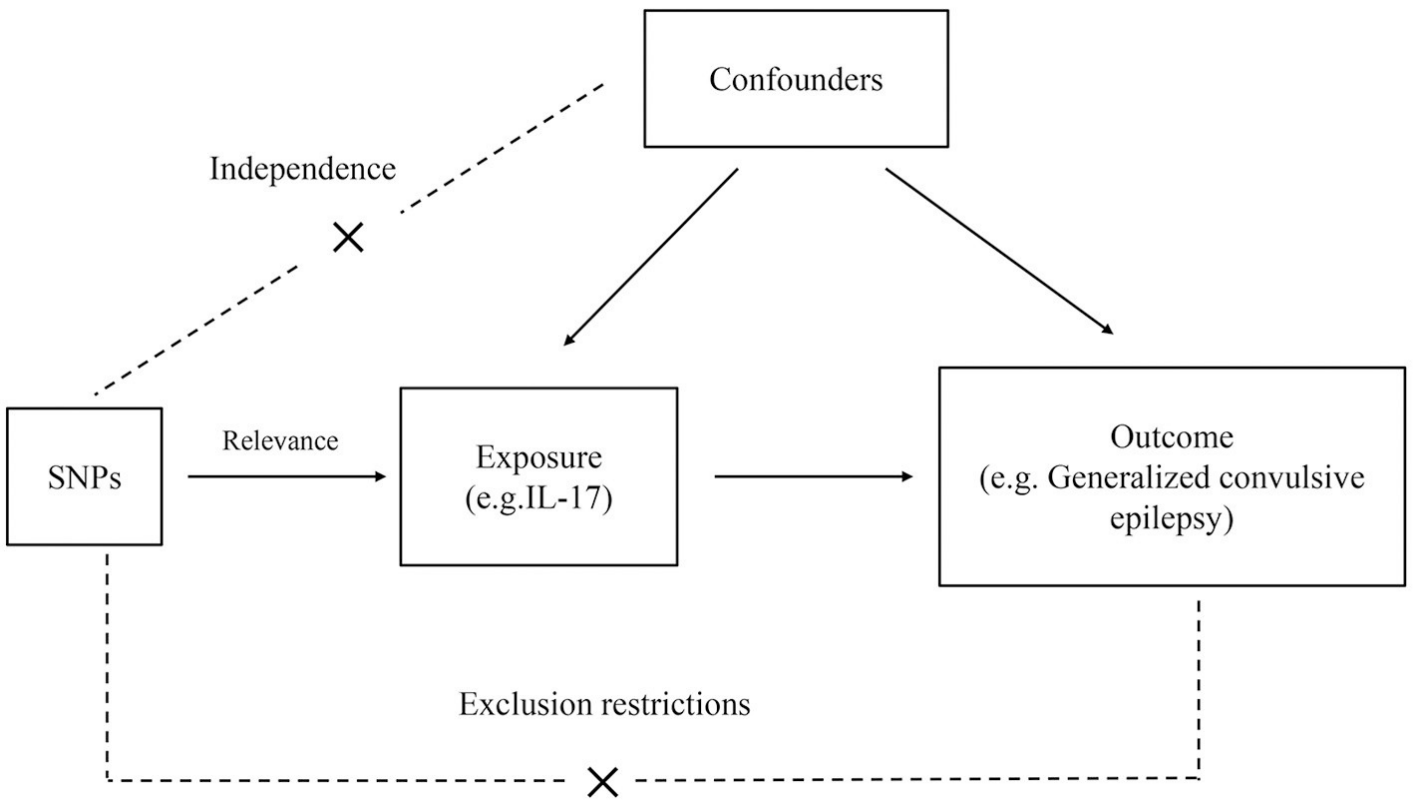
Flowchart of MR analysis in this study. SNPs, single nucleotide polymorphisms.

The general study design is illustrated in Figure 1. The genome-wide association study (GWAS) summary data on cytokines and epilepsy were derived from published studies; therefore, no additional ethical clearance was required. Comprehensive GWAS summary statistics are available from the GWAS Catalog server (https://www.ebi.ac.uk/gwas/home). There are no participants in this study who are not of European ancestry. To implement Mendelian randomization, three main assumptions must be met: (1) IVs strongly correlate with exposure. (2) IVs were independent of unidentified confounders between exposure and outcome. (3) IVs are only predicted to affect outcomes through exposure (6).

### 2.2. The selection of data sources and instruments

Data on circulating cytokines were extracted from 8,293 individuals, including 41 cytokines (7). An overview of the detailed GWAS data on the cytokines utilized in the MR analyses is presented in Table 1. GWAS summary statistics on epilepsy were obtained from the United Kingdom Biobank (UKB), involving 290 epilepsy cases and 4,56,058 European controls, and were adjusted for covariates using a generalized linear mixed model (GLMM)-based method named fastGWA-GLMM.

**Table 1:** Sample size for each cytokine analyzed in this study acquired from the GWAS.

**Cytokines**	**Abbreviation**	**Sample size**	**Number**
Cutaneous T-cell attracting (CCL27)	CTACK	3,631	GCST004420
Beta nerve growth factor	βNGF	3,531	GCST004421
Vascular endothelial growth factor	VEGF	7,118	GCST004422
Macrophage migration inhibitory factor (glycosylation-inhibiting factor)	MIF	3,494	GCST004423
TNF-related apoptosis inducing ligand	TRAIL	8,186	GCST004424
Tumor necrosis factor-beta	TNFβ	1,559	GCST004425
Tumor necrosis factor-alpha	TNFα	3,454	GCST004426
Stromal cell-derived factor-1 alpha (CXCL12)	SDF1α	5,998	GCST004427
Stem cell growth factor beta	SCGFβ	3,682	GCST004428
Stem cell factor	SCF	8,290	GCST004429
Interleukin-16	IL-16	3,483	GCST004430
Regulated on Activation, Normal T Cell Expressed and Secreted (CCL5)	RANTES	3,421	GCST004431
Platelet-derived growth factor BB	PDGFbb	8,293	GCST004432
Macrophage inflammatory protein-1β (CCL4)	MIP1β	8,243	GCST004433
Macrophage inflammatory protein-1α (CCL3)	MIP1α	3,522	GCST004434
Monokine induced by interferon-gamma (CXCL9)	MIG	3,685	GCST004435
Macrophage colony-stimulating factor	MCSF	840	GCST004436
Monocyte specific chemokine 3 (CCL7)	MCP3	843	GCST004437
Monocyte chemotactic protein-1 (CCL2)	MCP1	8,293	GCST004438
Interleukin-12p70	IL-12p70	8,270	GCST004439
Interferon gamma-induced protein 10 (CXCL10)	IP10	3,685	GCST004440
Interleukin-18	IL-18	3,636	GCST004441
Interleukin-17	IL-17	7,760	GCST004442
Interleukin-13	IL-13	3,557	GCST004443
Interleukin-10	IL-10	7,681	GCST004444
Interleukin-8 (CXCL8)	IL-8	3,526	GCST004445
Interleukin-6	IL-6	8,189	GCST004446
Interleukin-1 receptor antagonist	IL1ra	3,638	GCST004447
Interleukin-1-beta	IL-1β	3,309	GCST004448
Hepatocyte growth factor	HGF	8,292	GCST004449
Interleukin-9	IL-9	3,634	GCST004450
Interleukin-7	IL-7	3,409	GCST004451
Interleukin-5	IL-5	3,364	GCST004452
Interleukin-4	IL-4	8,124	GCST004453
Interleukin-2 receptor, alpha subunit	IL2rα	3,677	GCST004454
Interleukin-2	IL-2	3,475	GCST004455
Interferon-gamma	IFN-γ	7,701	GCST004456
Growth regulated oncogene-α (CXCL1)	GROα	3,505	GCST004457
Granulocyte colony-stimulating factor	GCSF	7,904	GCST004458
Basic fibroblast growth factor	bFGF	7,565	GCST004459
Eotaxin (CCL11)	Eotaxin	8,153	GCST004460

The threshold for genome-wide significance was *P* < 5 × 10^−6^ to avoid the presence of false positive IVs. In linkage disequilibrium, single nucleotide polymorphisms (SNPs) with the lowest *p*-values were retained as independent after pruning all SNPs in linkage disequilibrium LD (*r*2 < 0.001 in the European 1,000 G reference panel) (8). After harmonizing the selected SNPs with the outcome data, we selected all 41 systemic inflammation regulators. To ensure that there was no weak instrumental variable bias, we confirmed that the F-statistic was higher than 10, indicating that the study was strong enough to avoid bias (9).

### 2.3. Statistical analysis

This bidirectional MR study aimed to investigate the association between cytokine levels and epilepsy risk and vice versa. The primary analysis utilized the inverse variance-weighted (IVW) method, which estimated the combined causal effects of each SNP. Additionally, MR-Egger and weighted median analyses were used as complementary methods (10). Several sensitivity analyses were performed to ensure the robustness of the results. Furthermore, Cochran's Q test was used to evaluate the heterogeneity between the causal effects of different genetic variants, with a significance level of a *P*-value of < 0.05 (11). Additionally, the MR-PRESSO method was used to detect and remove possible outliers to obtain an unbiased causal estimate (12). Furthermore, a leave-one-out sensitivity analysis was conducted to assess the overall stability.

If the results of the IVW method were statistically significant (*P* < 0.05), the results were considered positive, even if the other methods did not reach statistical significance and no pleiotropy or heterogeneity was observed, provided that the beta values of the other methods were in the same direction (13). When horizontal pleiotropy was observed but not heterogeneity, the MR-Egger method was preferred; however, the weighted median method was selected when heterogeneity was observed but not pleiotropy. Alternatively, the multiplicative random effects inverse variance weighting (mre-IVW) method was employed for the analysis.

Analyses were conducted using the TwoSampleMR (version 0.5.6) and MR-PRESSO (version 1.0) packages in R (version 4.2.1).

## 3. Result

This study observed a bidirectional association between cytokine levels and epilepsy.

**Figure 2:**
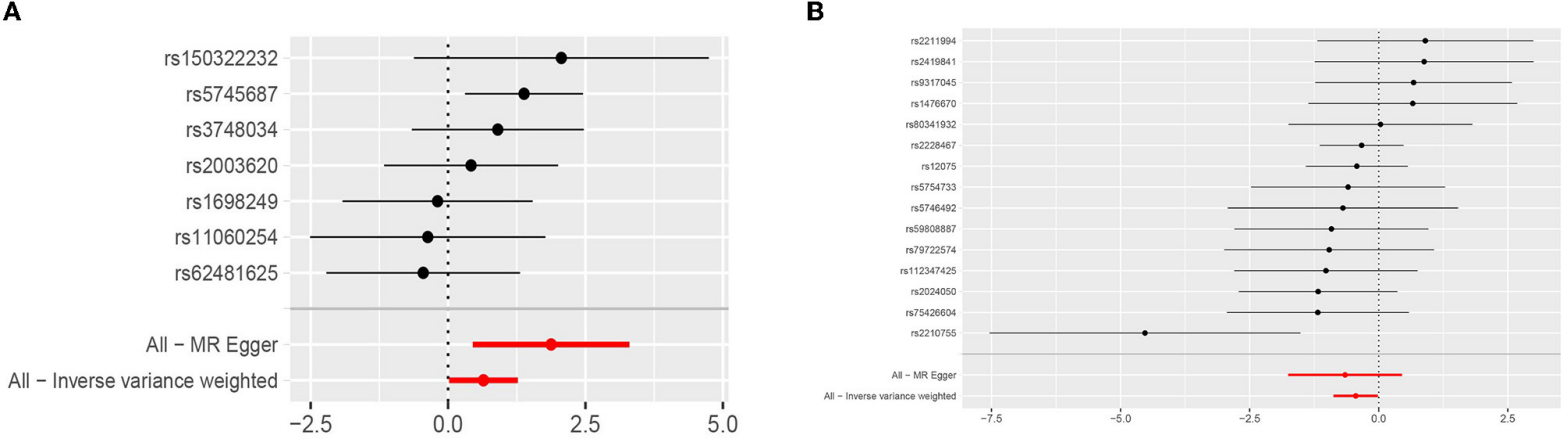
**(A)** Forest plots for the exposure of HGF. **(B)** Forest plots for the exposure of CCL11.

Genetically predicted cytokines associated with epilepsy. Reduced eotaxin (CCL11) and elevated hepatocyte growth factor (HGF) were associated with an increased risk of epilepsy (OR = 1.904, 95% CI = 1.019–3.561, *p* = 0.044; OR = 0.641, 95% CI = 0.417–0.984, *p* = 0.042) when the IVW method was used. Although elevated Interleukin-18 (IL-18) levels were associated with a decreased risk of epilepsy, the efficacy of the assessment may have been lower because the results were not significant (*P* > 0.05) when using the IVW method, thus necessitating the use of the MR-Egger method (OR = 0.482, 95% CI = 0.251–0.927, *p* = 0.046). Cochran's Q test, MR-Egger intercept, and MR-PRESSO global test analyses failed to detect heterogeneity and horizontal pleiotropy in the primary analysis (all *P* > 0.05). In addition, no single SNP significantly affected the overall effect of cytokines on epilepsy in the IVW in the leave-one-out sensitivity analysis. Figure 2 shows the forest plots of the above results.

**Figure 3:**
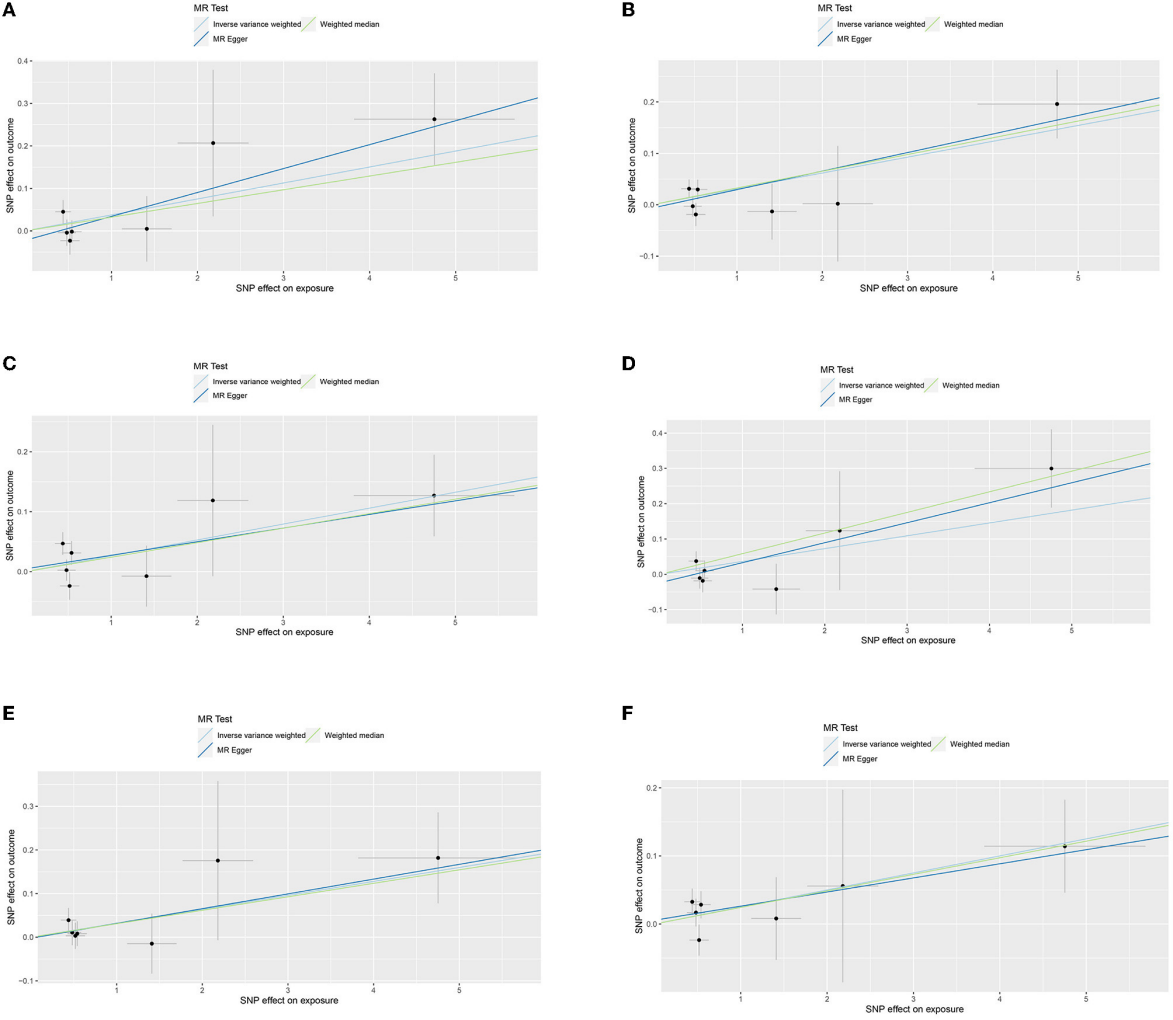
**(A)** Scatter plot for the outcome of CCL3. **(B)** Scatter plot for the outcome of IL-12p70. **(C)** Scatter plot for the outcome of IL-17. **(D)** Scatter plot for the outcome of IL-13. **(E)** Scatter plot for the outcome of IL-1ra. **(F)** Scatter plot for the outcome of bFGF.

**Figure 4:**
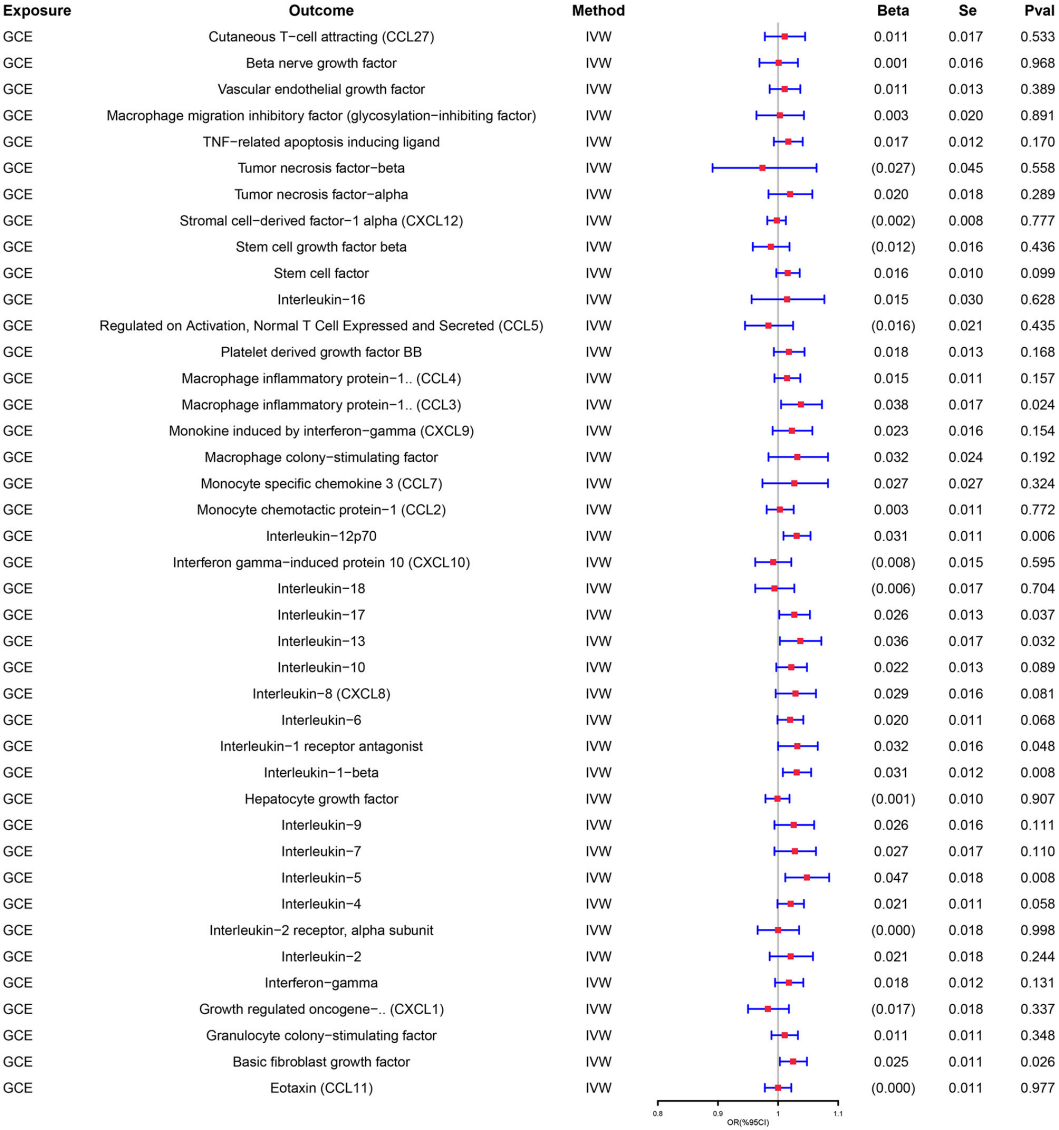
Association between genetically predicted GCE and the levels of cytokines.

**Figure 5:**
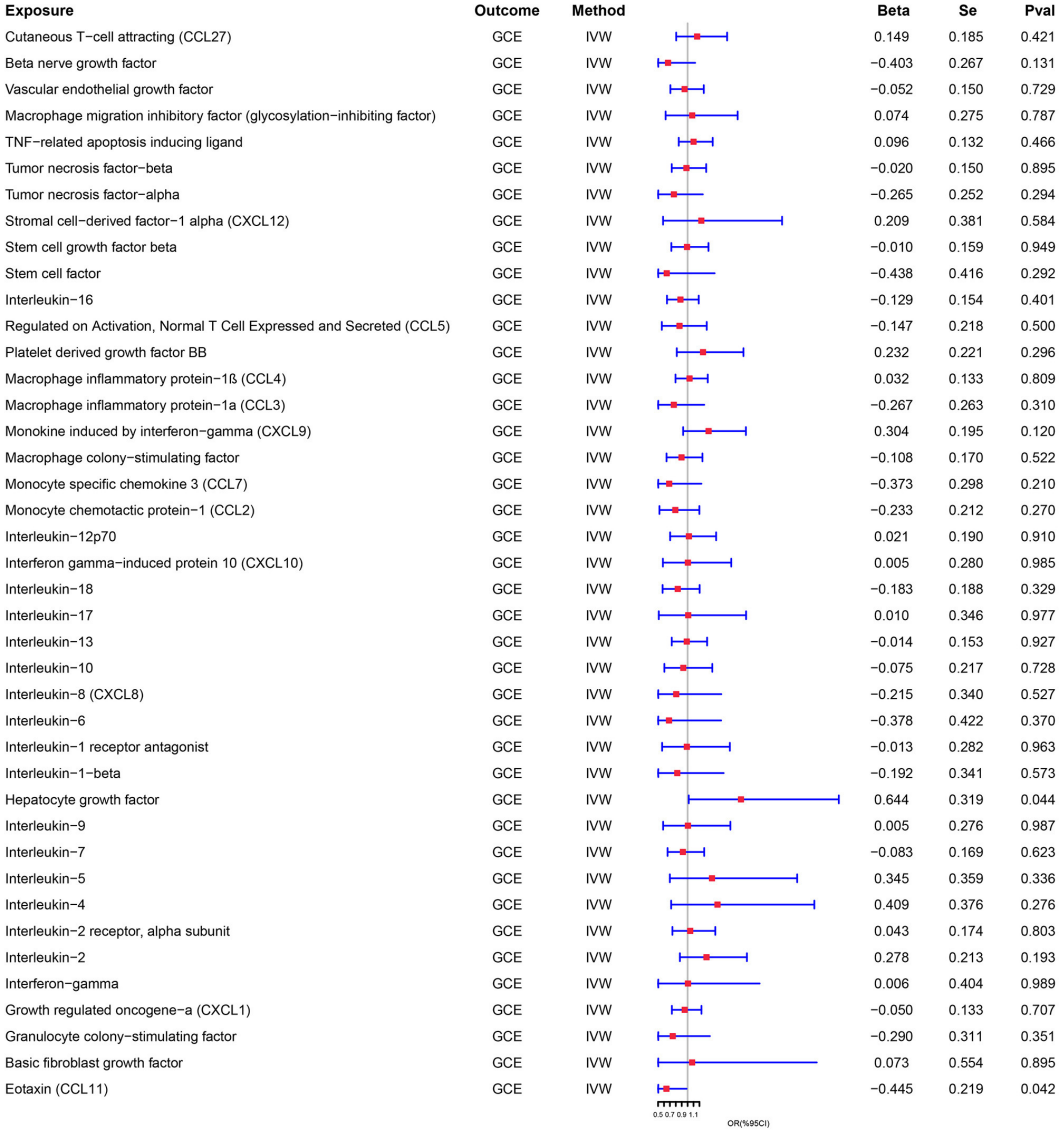
Association between genetically predicted the levels of cytokines and GCE.

Similarly, genetically predicted epileptogenesis was associated with elevated levels of multiple cytokines, such as macrophage inflammatory protein-1α (CCL3), Interleukin-12p70(IL-12p70), Interleukin-17(IL-17), Interleukin-1 receptor antagonist (IL-1ra), and basic fibroblast growth factor (bFGF) (OR = 1.038, 95% CI = 1.005–1.073, *p* = 0.024; OR = 1.031, 95% CI = 1.009–1.054, *p* = 0.006; OR = 1.027, 95% CI = 1.002–1.053, *p* = 0.037; OR = 1.037, 95% CI = 1.003–1.072, *p* = 0.032; OR = 1.032, 95% CI = 1.000–1.066, *p* = 0.048; OR = 1.025, 95% CI = 1.003–1.048, *p* = 0026). All results were significant when the MR was conducted using the IVW method as the primary analysis method. Furthermore, sensitivity tests using Cochran's Q test, MR-Egger intercept, and MR-PRESSO did not show significant results, confirming the reliability of the IVW method. In addition, the significant outcomes of MR and sensitivity analyses regarding predicting cytokines and GCE are displayed in Table 2. Figure 3 shows the scatter plots of the above results. The findings of the MR and sensitivity analyses between the cytokines and GCE are shown in Figures 4, 5, respectively.

**Table 2:** Results of the MR study testing causal association between systemic inflammatory regulators and risk of epilepsy.

**Cytokines**	**Number of SNPs**	**beta**	**OR (95% CI)**	**P**	**P for heterogeneity test**	**P for MR-Egger intercept**	**P for MR-PRESSO (0 outliers)**
**Hepatocyte growth factor**							
Inverse variance weighted	7	0.644	1.904 (1.019–3.561)	0.044	0.389	0.120	0.215
MR Egger	7	1.875	5.522 (1.568–27.132)	0.050	0.730		
Weighted median	7	0.685	1.983 (0.865–4.548)	0.106			
**Eotaxin (CCL11)**							
Inverse variance weighted	15	−0.445	0.641 (0.417–0.984)	0.042	0.346	0.694	0.061
MR Egger	15	−0.652	0.521 (0.173–1.569)	0.267	0.290		
Weighted median	15	−0.398	0.672 (0.386–1.167)	0.158			
**Interleukin-18 (IL-18)**							
Inverse variance weighted	16	−0.183	0.833 (0.576–1.203)	0.329	0.028	0.076	0.612
MR Egger	16	−0.728	0.482 (0.251–0.927)	0.046	0.090		
Weighted median	16	−0.332	0.718 (0.482–1.068)	0.102			
**Macrophage inflammatory protein-1**α **(CCL3)**							
Inverse variance weighted	7	0.038	1.038 (1.005–1.073)	0.024	0.486	0.327	0.089
MR Egger	7	0.056	1.058 (1.009–1.109)	0.066	0.510		
Weighted median	7	0.032	1.033 (0.987–1.081)	0.163			
**Interleukin-12p70(IL-12p70)**							
Inverse variance weighted	7	0.031	1.031 (1.009–1.054)	0.006	0.360	0.678	0.170
MR Egger	7	0.036	1.037 (1.003–1.072)	0.087	0.274		
Weighted median	7	0.033	1.033 (1.003–1.064)	0.033			
**Interleukin-17 (IL-17)**							
Inverse variance weighted	7	0.026	1.027 (1.002–1.053)	0.037	0.430	0.792	0.446
MR Egger	7	0.023	1.023 (0.985–1.063)	0.296	0.463		
Weighted median	7	0.024	1.024 (0.995–1.054)	0.100			
**Interleukin-13 (IL-13)**							
Inverse variance weighted	7	0.036	1.037 (1.003–1.072)	0.032	0.430	0.303	0.055
MR Egger	7	0.057	1.058 (1.009–1.110)	0.069	0.463		
Weighted median	7	0.058	1.060 (1.009–1.114)	0.021			
**Interleukin-1 receptor antagonist (IL-1ra)**							
Inverse variance weighted	7	0.032	1.032 (1.000–1.066)	0.048	0.593	0.914	0.100
MR Egger	7	0.034	1.034 (0.988–1.083)	0.204	0.472		
Weighted median	7	0.031	1.031 (0.989–1.075)	0.148			
**Basic fibroblast growth factor (bFGF)**							
Inverse variance weighted	7	0.025	1.025 (1.003–1.048)	0.026	0.597	0.707	0.190
MR Egger	7	0.021	1.021 (0.990–1.053)	0.240	0.489		
Weighted median	7	0.024	1.025 (0.995–1.055)	0.103			

## 4. Discussion

As one of the most common neurological disorders, the prevalence of epilepsy in the United States and Europe is 55 per 100,000 people annually, with approximately 65 million people affected worldwide (14). One study showed that GCE accounts for 23.8% of all epilepsy types (1). If not terminated in time, GCE has the potential to progress to convulsive status epilepticus, which significantly increases brain metabolism and causes irreversible brain tissue damage, ultimately leading to higher mortality and disability rates (15–17). Currently, the inability to assess the risk and susceptibility of GCE, lack of timely access to care, and poor patient compliance add to the challenges of treating GCE. Recent clinical and experimental studies have demonstrated a close relationship between epilepsy and inflammatory response, and the latter is a fundamental characteristic of specific areas of the brain (lesions with increased excitability) (18, 19). By screening 41 cytokines in the largest GWAS datasets available, we observed a bidirectional relationship between cytokines and GCE that can predict each other. Notably, the development of GCE can trigger an elevation in cytokines, including CCL3, IL-12p70, IL-17, IL-17, IL-1ra, and bFGF, and altered levels of cytokines, including CCL11, HGF, and IL-18, can cause changes in GCE risk. To the best of our knowledge, this is the largest and most comprehensive MR study exploring the causal relationship between inflammatory regulators and GCE.

The hepatocyte growth factor (HGF) plays a key role not only in the migration of interneurons from ganglionic protrusions to the cerebral cortex but also in the maintenance of neural circuit formation and cell survival (20). GABAergic neurons play an essential role in regulating excitatory neurotransmission (21). Reportedly, spontaneous seizures in adult mice after HGF supplementation contrasted with the reversal of epileptic behavior and stabilization of spontaneous EEG discharges when the HGF concentration was reduced (22). These studies demonstrated an inverse correlation between serum HGF levels and epilepsy, contradicting the findings of the present study that higher HGF levels corresponded with the occurrence of GCE. The different results may be because the samples of previous studies were animals, and specific types of epilepsy were not investigated. Moreover, the above animal experiments did not explain the specific types of seizures, which may have contributed to the difference between their research results and ours.

Some chemokine receptors, such as CCL3 and CCL11, are highly expressed in the hippocampus and are associated with epilepsy because they may promote the release of excitatory neurotransmitters (23, 24). By upregulating nicotinamide adenine dinucleotide phosphate oxidase-1, CCL11 promotes microglial migration, induces reactive oxygen species production, and enhances excitotoxic neuronal death (25). This may explain the mechanism underlying the proconvulsant effect of CCL11. Notably, a prospective study of 56 patients aged 0–16 showed that CCL11 concentrations were significantly higher in the refractory epilepsy group than in the control group (26). This discrepancy in the results of our study may be because of the insufficient number of patients in this prospective study and the inability of the participating population to represent patients of all ages.

IL-18 of the interleukin-1 family is produced almost exclusively by brain cells. Significant elevations in IL-18 concentrations were observed in both animal models and patients with epilepsy; however, no association was observed between epilepsy or seizure type and serum IL-18 levels (27). IL-18 gene amplification and elevated IL-18 concentrations have been observed in patients with temporal lobe epilepsy [29], suggesting that a genotype can increase inflammatory factors, leading to epilepsy. In the MR analysis of the present study, IL-18 was confirmed to cause epilepsy development only with the MR approach, which was less effective than the IVW approach. Furthermore, there was no significant change in seizure frequency in IL-18 knockout mice compared with wild-type mice (28). Therefore, the argument that IL-18 triggers the formation of GCE remains controversial.

Furthermore, IL-1β can exacerbate seizures by enhancing glutamate-mediated neurotransmission. IL-1ra, an endogenous antagonist of IL-1R, competes with IL-1β for binding to IL-1 receptors and inhibits seizures in the presence of IL-1ra overexpression. In addition, the intrahippocampal injection of exogenous IL-1ra reduced the incidence of epilepsy (29). The MR analysis in this study revealed that IL-1ra was present downstream of epilepsy (i.e., seizures resulted in significant changes in IL-1ra), and elevated IL-1ra levels may represent a protective mechanism of the organism. Therefore, altering the IL1RA/IL1β ratio by endogenous or exogenous supplementation or reduction may be a potential therapeutic target for GCE.

In this study, there was also a marked elevation in the levels of cytokines other than IL-1ra after epilepsy, including CCL3, IL-12p70, IL-13, IL-17, and bFGF.

Elevations in CC chemokine receptor 5 (CCR5) and CCL3 ligands of CCR5 are observed in epilepsy (30). Mouse animal models have demonstrated a central role for CCR5 and its ligands in mediating inflammation, suggesting that reduced levels of CCR5 may have neuroprotective and neurodegenerative effects (28). In a pilocarpine epilepsy model, status epilepticus promoted leukocyte 4 integrin-mediated leukocyte adhesion to vascular cell adhesion molecule-1, whereas antibodies against leukocyte 4 integrin can prevent seizure development and status epilepticus (31). It has also been observed that inflammation can also increase neuronal excitability and promote epilepsy or increase its susceptibility (3, 32). Therefore, based on the animal experiments described above, further studies regarding CCR5 and CCL3 as potential pathways for epilepsy treatment are warranted. However, multiple ligands can interact with CCR5, and much work remains to be done to determine optimal treatment options.

IL-12p70, a subunit of IL-12, is a biologically active cytokine secreted by monocytes, macrophages, and dendritic cells that plays an important role in inflammation and antitumor immunity, and also in the proliferation of NK cells, induction of cytotoxic T cell maturation, and increased toxicity (33, 34). This study demonstrated elevated IL-12p70 levels after GCE, suggesting that this cytokine is associated with secondary brain injury. Furthermore, studies have shown that GCE can induce an inflammatory response, resulting in increased serum high-mobility group box 1 (HMGB1) levels (35); in contrast, HMGB1 inactivation or receptor blockade relieves epilepsy (36), suggesting that GCE may be alleviated by targeting IL12p70 antagonists or their receptors.

IL-13, an anti-inflammatory cytokine, is involved in various physiological and pathological processes, such as cell proliferation, apoptosis, and inflammatory and immune reactions. In treating drug-resistant epilepsy, vagus nerve stimulation (VNS) is used (37). A previous study showed that in adult rats with seizures, IL-13 was significantly increased after VNS, and the frequency and duration of seizures were significantly lower than those in the group that did not undergo VNS implantation but had seizures (38). This suggests that VNS treatment of epilepsy may be achieved by upregulating anti-inflammatory factors such as IL-13. Considering the elevated IL-13 concentrations after seizures and its anti-inflammatory properties, further studies are warranted to monitor the therapeutic effects of GCE.

Currently, there is a lack of studies comparing IL-17 and GCE, with only references to other studies. IL-17 concentrations are higher in the serum and cerebrospinal fluid of patients with epilepsy (39, 40). Furthermore, IL-17 can be used as an independent marker for evaluating the severity of temporal lobe epilepsy and idiopathic generalized epilepsy (41). In addition, previous studies have shown that IL-17 can contribute to the development of epilepsy through various pathogenic mechanisms such as neuroinflammation, breakdown of the blood-brain barrier, abnormal neurotransmitter transmission, and abnormal ion channel activity. Therefore, we assumed that, in addition to being used to monitor disease severity, IL-17 is a potential anti-GCE target.

Furthermore, the basic fibroblast growth factor (bFGF) is widely distributed in peripheral tissues and the central nervous system and has significant neurotrophic and protective effects on neurons and glial cells. In animal experiments, the intracerebroventricular infusion of bFGF significantly reduced hippocampal neuronal loss and seizure frequency in rats with epilepsy (42). The post-epileptic cytokine elevation observed in this study may provide feedback on this protective mechanism. Therefore, promoting the synthesis of endogenous bFGF or supplementing exogenous bFGF through appropriate pathways provides a new avenue for treating GCE.

Despite the importance of this study, it has some limitations. These data only apply to European populations, and other ethnic groups may not benefit. The definition of GCE remains broad and unclear and may present inherent limitations in utilizing datasets for research. Nevertheless, the current understanding of seizure subtypes in epilepsy has room for improvement, and more comprehensive data are necessary to obtain a higher level of evidence. Moreover, because of the lack of categorized data on GCE patient characteristics, such as the age of onset, sex, and disease severity (e.g., seizure frequency and duration), it remains difficult to analyze by category according to the above indicators.

In conclusion, there was a causal link between the cytokines and GCE. Detecting significantly altered factor concentrations may be of great significance for screening GCE and predicting their occurrence. Moreover, available pharmacological treatments for GCE primarily focus on suppressing seizures. Subsequently, altering the concentration of these cytokines in the body through targeted anti-inflammatory therapy modifies the epileptogenic mechanism and prevents the recurrence and refractoriness of GCE. Therefore, reducing epilepsy-induced cognitive, motor, and behavioral impairments may become the key to new treatments.

## Data availability statement

The original contributions presented in the study are included in the article/supplementary material, further inquiries can be directed to the corresponding authors.

## Ethics statement

Ethical review and approval was not required for the study on human participants in accordance with the local legislation and institutional requirements. Written informed consent from the patients/participants or patients/participants' legal guardian/next of kin was not required to participate in this study in accordance with the national legislation and the institutional requirements.

## Author contributions

SW and TS drafted the article and made substantial contributions to the conception or design of the study. SP and YL made a substantial, direct, and intellectual contribution to the study. LC and MZ are in agreement to be accountable for all aspects of the study in ensuring that questions related to the accuracy or integrity of any part of the study are appropriately investigated and resolved. JW improved the data analysis after our subsequent rework. All authors have read, agreed to the final version of the manuscript, and made substantial contributions to the manuscript.

## References

[1] Sapkota S Kobau R Pastula DM Zack MM. People with epilepsy are diagnosed most often with unspecified epilepsy, followed by focal epilepsy, generalized convulsive epilepsy, and generalized nonconvulsive epilepsy-US MarketScan data, 2010-2015. Epilepsy Behav E&B. (2018) 79:244–6. 10.1016/j.yebeh.2017.11.004 29249447

[2] Willoughby JO Mackenzie L Medvedev A Hiscock JJ. Generalized convulsive epilepsy: possible mechanisms. J Clin Neurosci J Neurosurg Soc Au. (1999) 6:189–94. 10.1016/S0967-5868(99)90500-3 10835165

[3] Vezzani A Granata T. Brain inflammation in epilepsy: experimental and clinical evidence. Epilepsia. (2005) 46:1724–43. 10.1111/j.1528-1167.2005.00298.x 16302852

[4] Soltani Khaboushan A Yazdanpanah N Rezaei N. Neuroinflammation and proinflammatory cytokines in epileptogenesis. Mol Neurobiol. (2022) 59:1724–43. 10.1007/s12035-022-02725-6 35015252

[5] Burgess S Daniel RM Butterworth AS Thompson SG. Network Mendelian randomization: using genetic variants as instrumental variables to investigate mediation in causal pathways. Int J Epidemiol. (2015) 44:484–95. 10.1093/ije/dyu176 25150977 PMC4469795

[6] Smith GD Ebrahim S. 'Mendelian randomization': can genetic epidemiology contribute to understanding environmental determinants of disease? Int J Epidemiol. (2003) 32. 10.1093/ije/dyg070 12689998

[7] Ahola-Olli AV Würtz P Havulinna AS Aalto K Pitkänen N Lehtimäki T. Genome-wide association study identifies 27 loci influencing concentrations of circulating cytokines and growth factors. Am J Hum Genet. (2017) 100:40–50. 10.1016/j.ajhg.2016.11.007 27989323 PMC5223028

[8] Georgakis MK De Lemos JA Ayers C Wang B Björkbacka H Pana TA. Association of circulating monocyte chemoattractant protein-1 levels with cardiovascular mortality: a meta-analysis of population-based studies. JAMA Cardiol. (2021) 6:587–92. 10.1001/jamacardio.2020.5392 33146689 PMC8111478

[9] Bowden J Del Greco M Minelli F Davey Smith C Sheehan GNA Thompson JR. Assessing the suitability of summary data for two-sample Mendelian randomization analyses using MR-Egger regression: the role of the I2 statistic. Int J Epidemiol. (2016) 45:1961–74. 10.1093/ije/dyw220 27616674 PMC5446088

[10] Larsson SC Scott RA Traylor M Langenberg CC Hindy G Melander O. Type 2 diabetes, glucose, insulin, BMI, and ischemic stroke subtypes: Mendelian randomization study. Neurology. (2017) 89:454–60. 10.1212/WNL.0000000000004173 28667182 PMC5539736

[11] Bowden J Del Greco M Minelli F Davey Smith C Sheehan GN Thompson J. A framework for the investigation of pleiotropy in two-sample summary data Mendelian randomization. Stat Med. (2017) 36:1783–802. 10.1002/sim.7221 28114746 PMC5434863

[12] Verbanck M Chen CY Neale B Do R. Detection of widespread horizontal pleiotropy in causal relationships inferred from Mendelian randomization between complex traits and diseases. Nature Gen. (2018) 50, 693–698. 10.1038/s41588-018-0099-7 29686387 PMC6083837

[13] Chen X Kong J Diao X Cai J Zheng J Xie W. Depression and prostate cancer risk: a Mendelian randomization study. Cancer Med. (2020) 9:9160–7. 10.1002/cam4.3493 33027558 PMC7724297

[14] Eimon PM Ghannad-Rezaie M De Rienzo G Allalou A Wu Y Gao M. Brain activity patterns in high-throughput electrophysiology screen predict both drug efficacies and side effects. Nature Commun. (2018) 9:219. 10.1038/s41467-017-02404-4 29335539 PMC5768723

[15] Damian MS Ben-Shlomo Y Howard R Harrison DA. Admission patterns and survival from status epilepticus in critical care in the UK: an analysis of the intensive care national audit and research centre case mix programme database. Eur J Neurol. (2020) 27:557–64. 10.1111/ene.14106 31621142

[16] Dhakar MB Thurman DJ Haider HA Rodriguez AR Jette N. Thirty-day readmission after status epilepticus in the United States: Insights from the nationwide readmission database. Epilepsy Res. (2020) 165:106346. 10.1016/j.eplepsyres.2020.106346 32521438

[17] Alkhachroum A Der-Nigoghossian CA Rubinos C Claassen J. Markers in status epilepticus prognosis. J Clin Neurophysiol Am Electroencephal Soc. (2020) 37:422–8. 10.1097/WNP.0000000000000761 32890064 PMC7864547

[18] Iori V Frigerio F Vezzani A. Modulation of neuronal excitability by immune mediators in epilepsy. Curr Opin Pharmacol. (2016) 26:118–23. 10.1016/j.coph.2015.11.002 26629681 PMC4716878

[19] Vezzani A French J Bartfai T Baram TZ. The role of inflammation in epilepsy. Nature Reviews Neurology. (2011) 7:31–40. 10.1038/nrneurol.2010.178 21135885 PMC3378051

[20] Powell EM Mars WM Levitt P. Hepatocyte growth factor/scatter factor is a motogen for interneurons migrating from the ventral to dorsal telencephalon. Neuron. (2001) 30:79–89. 10.1016/S0896-6273(01)00264-1 11343646

[21] Wonders CP Anderson SA. The origin and specification of cortical interneurons. Nat Rev Neurosci. (2006) 7:687–96. 10.1038/nrn1954 16883309

[22] Bae MH Bissonette GB Mars WM Michalopoulos GK Achim CL Depireux DA. Hepatocyte growth factor (HGF) modulates GABAergic inhibition and seizure susceptibility. Exp Neurol. (2010) 221:129–35. 10.1016/j.expneurol.2009.10.011 19853606 PMC2812579

[23] Aulická S Ceská K Šána J Siegl F Brichtová E Ošlejšková H. Cytokine-chemokine profiles in the hippocampus of patients with mesial temporal lobe epilepsy and hippocampal sclerosis. Epilepsy Res. (2022) 180, 106858. 10.1016/j.eplepsyres.2022.106858 35026708

[24] Cerri C Caleo M Bozzi Y. Chemokines as new inflammatory players in the pathogenesis of epilepsy. Epilepsy Res. (2017) 136:77–83. 10.1016/j.eplepsyres.2017.07.016 28780154

[25] Teixeira AL Gama CS Rocha NP Teixeira MM. Revisiting the role of eotaxin-1/CCL11 in psychiatric disorders. Front Psychiatry. (2018) 9:241. 10.3389/fpsyt.2018.00241 29962972 PMC6010544

[26] Gakharia T Bakhtadze S Lim M Khachapuridze N Kapanadze N. Alterations of plasma pro-inflammatory cytokine levels in children with refractory epilepsies. Children. (2022) 9:1506. 10.3390/children9101506 36291442 PMC9600205

[27] Mochol M Taubøll E Aukrust P Ueland T Andreassen OA. Interleukin 18 (IL-18) and its binding protein (IL-18BP) are increased in patients with epilepsy suggesting low-grade systemic inflammation. Seizure. (2020) 80:221–5. 10.1016/j.seizure.2020.05.018 32659652

[28] Louboutin JP Strayer DS. Relationship between the chemokine receptor CCR5 and microglia in neurological disorders: consequences of targeting CCR5 on neuroinflammation, neuronal death and regeneration in a model of epilepsy. CNS Neurol Disord Drug Targets. (2013) 12:815–29. 10.2174/18715273113126660173 24047524

[29] Vezzani A Moneta D Conti M Richichi C Ravizza T De Luigi A. Powerful anticonvulsant action of IL-1 receptor antagonist on intracerebral injection and astrocytic overexpression in mice. Proceed Nati Acad Sci USA. (2000) 97:11534–9. 10.1073/pnas.190206797 11016948 PMC17235

[30] Van Gassen KL De Wit M Koerkamp MJ Rensen MG Van Rijen PC Holstege FC. Possible role of the innate immunity in temporal lobe epilepsy. Epilepsia. (2008) 49:1055–65. 10.1111/j.1528-1167.2007.01470.x 18076643

[31] Fabene PF Navarro Mora G Martinello M Rossi B Merigo F Ottoboni L. A role for leukocyte-endothelial adhesion mechanisms in epilepsy. Nat Med. (2008) 14:1377–83. 10.1038/nm.1878 19029985 PMC2710311

[32] Lee TS Mane S Eid T Zhao H Lin A Guan Z. Gene expression in temporal lobe epilepsy is consistent with increased release of glutamate by astrocytes. Mol Med. (2007) 13:1–3. 10.2119/2006-00079.Lee 17515952 PMC1869627

[33] De Vries JE Zurawski G. Immunoregulatory properties of IL-13: its potential role in atopic disease. Int Arch All Immunol. (1995) 106:175–9. 10.1159/000236842 7888780

[34] Rachman Rinaldi I. Coagulopathy in dengue infection and the role of interleukin-6. Acta Med Indonesiana. (2006) 38:105–8. 16799214

[35] Nass RD Wagner M Surges R Holdenrieder S. Time courses of HMGB1 and other inflammatory markers after generalized convulsive seizures. Epilepsy Res. (2020) 162:106301. 10.1016/j.eplepsyres.2020.106301 32126476

[36] Fu L Liu K Wake H Teshigawara K Yoshino T Takahashi H. Therapeutic effects of anti-HMGB1 monoclonal antibody on pilocarpine-induced status epilepticus in mice. Sci Rep. (2017) 7:1179. 10.1038/s41598-017-01325-y 28446773 PMC5430706

[37] Johnson RL Wilson CG. A review of vagus nerve stimulation as a therapeutic intervention. J Inflamm Res. (2018) 11:203–13. 10.2147/JIR.S163248 29844694 PMC5961632

[38] Qi R Wang M Zhong Q Wang L Yang X Huang B. Chronic vagus nerve stimulation (VNS) altered IL-6, IL-1β, CXCL-1 and IL-13 levels in the hippocampus of rats with LiCl-pilocarpine-induced epilepsy. Brain Res. (2022) 1780:147800. 10.1016/j.brainres.2022.147800 35074405

[39] Mao LY Ding J Peng WF Ma Y Zhang YH Fan W. (2013). Interictal interleukin-17A levels are elevated and correlate with seizure severity of epilepsy patients. Epilepsia, 54, e142–e145. 10.1111/epi.12337 23944193

[40] Montes M Zhang X Berthelot L Laplaud DA Brouard S Jin J. Oligoclonal myelin-reactive T-cell infiltrates derived from multiple sclerosis lesions are enriched in Th17 cells. Clinical Immunol. (2009) 130:133–44. 10.1016/j.clim.2008.08.030 18977698 PMC6961709

[41] Wang Y Wang D Guo D. Interictal cytokine levels were correlated to seizure severity of epileptic patients: a retrospective study on 1218 epileptic patients. J Transl Med. (2015) 13:378. 10.1186/s12967-015-0742-3 26626560 PMC4666166

[42] Liu Z D'Amore PA Mikati M Gatt A Holmes GL Neuroprotective effect of chronic infusion of basic fibroblast growth factor on seizure-associated hippocampal damage. Brain Res. (1993). 626:335–8. 10.1016/0006-8993(93)90598-H 8281447

